# GSK3β inactivation promotes the oncogenic functions of EZH2 and enhances methylation of H3K27 in human breast cancers

**DOI:** 10.18632/oncotarget.11008

**Published:** 2016-08-02

**Authors:** How-Wen Ko, Heng-Huan Lee, Longfei Huo, Weiya Xia, Cheng-Chieh Yang, Jennifer L. Hsu, Long-Yuan Li, Chien-Chen Lai, Li-Chuan Chan, Chien-Chia Cheng, Adam M. Labaff, Hsin-Wei Liao, Seung-Oe Lim, Chia-Wei Li, Yongkun Wei, Lei Nie, Hirohito Yamaguchi, Mien-Chie Hung

**Affiliations:** ^1^ Department of Molecular and Cellular Oncology, The University of Texas MD Anderson Cancer Center, Houston, Texas 77030, USA; ^2^ The University of Texas Graduate School of Biomedical Sciences at Houston, Houston, Texas 77030, USA; ^3^ Department of Thoracic Medicine, Chang Gung Memorial Hospital, Chang Gung University College of Medicine, Taoyuan 333, Taiwan; ^4^ Department of Dentistry, School of Dentistry, National Yang-Ming University, Taipei 112, Taiwan; ^5^ Center for Molecular Medicine and Graduate Institute of Cancer Biology, China Medical University, Taichung 404, Taiwan; ^6^ Department of Life Sciences, National Chung Hsing University, Taichung 402, Taiwan; ^7^ Institute of Molecular Biology, National Chung Hsing University, Taichung 402, Taiwan; ^8^ Department of Biotechnology, Asia University, Taichung 413, Taiwan

**Keywords:** EZH2, GSK3β, H3K27me3, cancer, phosphorylation

## Abstract

During the process of tumorigenesis, inactivation of tumor suppressors is a critical step. EZH2, a histone methyltransferase, promotes cell growth and migration through catalyzing trimethylation of histone H3 at Lys 27 (H3K27me3) and plays an important role in tumorigenesis. Its expression can be controlled by phosphorylation. However, the regulation of EZH2 activity by tumor suppressor kinase is not well understood. In this study, we show that glycogen synthase kinase 3 beta (GSK3β) negatively regulates H3K27 trimethylation. We also validate that GSKβ physically interacts with EZH2, and their interaction occurs in the cytosol. GSK3β phosphorylates EZH2 at Ser363 and Thr367 *in vitro*, and activating GSK3β upregulates Thr367 phosphorylation*in vivo*. Cells expressing GSK3β-non-phosphorylatable mutant EZH2 have higher H3K27 trimethylation and enhanced ability of cell migration and anchorage-independent growth. Inactivation of GSK3β as measured by its phosphorylation at Ser9 is positively correlated with higher level of H3K27 trimethylation in tumor tissues from breast cancer patients. Our study indicated that GSK3β phosphorylates EZH2 at Ser363 and Thr367, resulting in reduced H3K27 trimethylation and biological activity of EZH2 in breast cancer.

## INTRODUCTION

Epigenetic regulation, including histone modification, is an important mechanism that mediates gene expression by modifying the chromatin structure without changing the DNA sequences and is also essential for cell differentiation and tissue development. Aberrations of this regulation can alter gene expression, induce neoplastic cell transformation, and cause tumor growth [1]. Enhancer of zeste homolog 2 (EZH2), a histone methyltransferase and key player in epigenetics, serves as the enzymatic core subunit of polycomb repressive complex 2 (PRC2). The EZH2/PRC2 complex catalyzes the trimethylation of histone H3 at lysine 27 (H3K27) on the promoter of its target genes, such as HOX genes, to recruits other polycomb group proteins and factors, to silence gene expression [2, 3]. The histone methyltransferase activity of EZH2 requires its association with other PRC2 components, primarily suppressor of zeste 12 (SUZ12) and embryonic ectoderm development (EED) [4]. EZH2 and other polycomb group proteins play critical roles in many biological processes, like germline development, X-chromosome inactivation, and maintenance of stem cell properties [5].

In addition, EZH2 has been reported to play an important role in tumorigenesis. Evidence has shown that EZH2 is highly expressed in many types of solid tumors, including breast cancer, and its higher expression is associated with aggressive disease and poor outcome [6–8]. Several studies have also demonstrated that EZH2 contributes to tumorigenesis through promoting malignant transformation, cell proliferation, invasion and migration [8–12]. In mammary epithelial cells, overexpression of EZH2 enhances anchorage-independent growth and cell invasion and EZH2-mediated cell invasion requires its enzymatic domain, Su(var)3-9, enhancer of zeste, trithorax (SET) domain [8]. In breast tumor initiating cells, increased EZH2 expands cell population through repressing *RAD51* expression, and knockdown of EZH2 reduces the repression of *RAD51* transcription by decreasing H3K27 trimethylation [9]. Furthermore, in many types of cancer cells, EZH2 mediates cell proliferation, invasion and migration by epigenetically repressing tumor suppressor gene expressions through trimethylating H3K27 [10–12]. These studies have strongly linked EZH2 to oncogenesis and suggested that the oncogenic role of EZH2 partly relies on its capacity of catalyzing H3K27 trimethylation to repress specific gene transcription [13].

In the process of tumorigenesis, inactivation or loss of function of tumor suppressors is a crucial step. Several kinases such as AKT1, CDK1/2, p38α, and JAK2 have been reported to modulate EZH2 activity through posttranslational modifications [14–22]. For example, the first discovery of this regulation is through AKT1 phosphorylation, in which EZH2 is phosphorylated at Ser21 which subsequently alters its affinity for histone H3 in breast cancer [14]. This phosphorylation was later found to enhance the enzymatic activity of EZH2 in regulating non-histone substrates, such as androgen receptor and STAT3, in different cancer types [23, 24]. In addition, CDK2-mediated Thr416 phosphorylation augments cell migration, invasion, and tumor growth, and higher phosphorylation is correlated with poorer survival in triple-negative breast cancer patients [19]. However, the regulation of EZH2 activity by tumor suppressor kinase in cancer remains unclear. Interestingly, we noticed that the EZH2 amino acid sequence contains several glycogen synthase kinase 3 beta (GSK3β) phosphorylation motifs (Ser/Thr-X-X-X-Ser/Thr, where X represents any amino acid) [25], and GSK3β and EZH2 interaction has been shown in nasopharyngeal cancer with unknown consequence [26]. Together, these findings suggest that EZH2 might be regulated by GSK3β. GSK3β, a serine/threonine kinase, was initially identified as a critical mediator in glycogen metabolism, and has later been shown to be involved in diverse cellular processes, such as transcription, protein synthesis, cell cycle/proliferation, and microtube dynamics, through directly phosphorylation of a wide range of substrates (eIF2B, cycline D1, Tau, Snail and Mcl-1) [27–29]. Unlike other kinase, GSK3β is active at resting state but becomes inactive upon extracellular stimuli. The activity of GSK3β is controlled by site-specific phosphorylations, and Ser9 phosphorylation is probably the most well-known regulation which inhibits its activity [25]. Several proteins, such as protein kinase A, Akt, p90 ribosomal S6 kinase (p90RSK), and p70 ribosomal S6 kinase (p70S6K), inactivate GSK3β via this modification. GSK3β also participates in neoplastic transformation and tumor development [30]. Since GSK3β negatively regulates many oncoproteins and cell cycle regulators, it may function as a tumor suppressor. For example, GSK3β has been shown to phosphorylate and degrade β-catenin, and is a well-known, negative mediator of the canonical Wnt/β-catenin signaling pathway [31]. GSK3β also inhibits cell proliferation through regulation of Mcl-1 degradation [29]. In breast cancer cells, GSK3β suppresses epithelial-mesenchymal transition by control of Snail stabilization [28]. Moreover, overexpression or activation of GSK3β suppresses anchorage-independent cell growth in different types of cancer cells, whereas inactivation of GSK3β by expressing kinase deficient mutant promotes cell transformation and mammary tumorigenicity [32–34]. These studies support GSK3β's tumor suppressor role and reinforce its importance in tumorigenesis. However, little is known about the role of GSK3β in epigenetic regulation during tumor development.

In this study, we showed that GSK3β physically associated with and phosphorylated EZH2. Furthermore, this regulation suppressed EZH2 oncogenic functions and EZH2 enzymatic activity (trimethylation of H3K27) is inversely associated with GSK3β activity in tumor tissues from human breast cancer patients.

## RESULTS

### GSK3β negatively regulates H3K27 trimethylation

**Figure 1:**
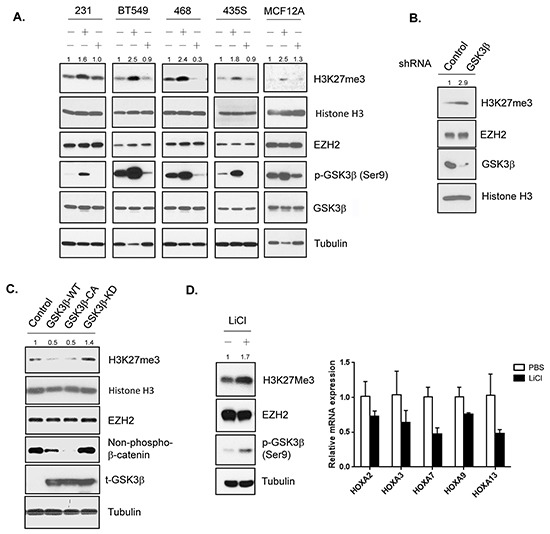
GSK3β downregulates H3K27 trimethylation **A.** MDA-MB-231, BT549, MDA-MB-468, MDA-MB-435S, and MCF12A cells were treated with lithium chloride (LiCl), staurosporine (STS) as indicated. Cell lysates were subjected to western blot analysis with the indicated antibodies. The intensities of H3K27me3 bands from treated cells were compared to those from untreated cells and the relative ratios are shown. **B.** Cells infected with lentiviruses expressing control or GSK3β shRNA were lyzed and analyzed by immunoblot with antibodies against indicated proteins. Relative intensitis of H3K27me3 bands are shown. **C.** Equal amounts of lysates from cells transfected with the plasmids encoding wild-type (WT), constitutively active (CA), kinase-dead (KD) GSK3β or empty vector control were analyzed by Western blot using antibodies against specific proteins. Relative intensities of H3K27me3 bands are shown, normalized to the intensity of H3K27me3 band from cells transfected with control plasmid. **D.** Left: Western blot analysis of cells treated with PBS or LiCl with the indicated antibodies. Lysates were immunobloted with the indicated antibodies. Relative intensities of H3K27me3 bands are shown. Right: qPR-PCR analysis of relative mRNA expression of *HOXA* families genes in lysates from MDA-MB-231 cells treated with PBS or LiCl. Data are expressed as mean ± s.d. (n = 3).

To investigate the regulation of EZH2 by GSK3β, we first determined whether alteration of GSK3β activity affects H3K27 trimethylation. We found that inhibition of GSK3β by lithium chloride, a GSK3β inhibitor, increased H3K27 trimethylation expression in multiple breast cancer cell lines including MDA-MB-231, BT549, MDA-MB-468, and MDA-MB-435S cells and mammary epithelial cells, MCF12A, and conversely, enhancing GSK3β activity using the anticancer drug staurosporine in MDA-MB-468, MDA-MB-435S and BT549 reduced the H3K27 trimethylation level (Figure 1A). There was no change in EZH2 level. Consistently, knockdown of GSK3β by small hairpin RNA enhanced trimethylation of H3K27 in HeLa cells (Figure 1B). GSK3β can phosphorylate and degrade β-catenin [31], and inhibition of GSK3β increases non-phospho-β-catenin level [35]. Thus, we used the level of non-phospho-β-catenin as a determinant of GSK3β activity. We found that exogenous expression of the wild type or constitutively active form of GSK3β, as indicated by the reduced level of non-phospho-β-catenin, decreased H3K27 trimethylation expression (Figure 1C). We also examined the expression of *HOXA* family genes, known to be repressed by EZH2 [5], and found that lithium chloride downregulated the expression of many of *HOXA* genes (Figure 1D). These results suggested that inactivation of GSK3β upregulates the H3K27 trimethylation resulting in reduced expression of EZH2-targeted genes.

### GSK3β interacts with EZH2

**Figure 2:**
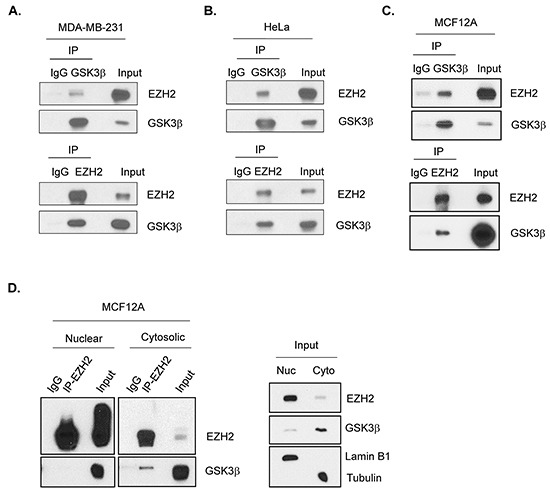
GSK3β interacts with EZH2 **A.** MDA-MB-231 cells were transfected with plasmids encoding Flag-EZH2. Cell lysates were immunoprecipitated with GSK3β (upper panel) or Flag (lower panel) antibodies, followed by Westeren blot analysis to detect EZH2 and GSK3β as indicated. Immunocipitation with immunoglobulin (IgG) served as a control. **B.** HeLa cells were transfected with plasmids encoding HA-GSK3β. Cell lysates were immunoprecipitated with HA (upper panel) or EZH2 (lower panel) antibodies, followed by Westeren blot analysis with antibodies against EZH2 and GSK3β. **C.** Cell lysates from MCF12A cells were immunoprecipitated by either GSK3β (upper panel) or EZH2 (lower panel) antibodies, then immunoblotted with indicated antibodies. **D.** MCF12A cells were lysed and followed by cellular fractionation. Nuclear (Nuc) and cytosolic (Cyto) fractions were immunoprecipitated with EZH2 antibody and immunoblotted with antibodies against EZH2 and GSK3β. Lamin B1 and Tubulin were used as markers for nuclear and cytosolic fractions, respectively.

Since altering GSK3β activity affects the trimethylation of H3K27 and the expression of EZH2-targeted genes, and GSK3β-EZH2 interaction has been shown in nasopharyngeal cancer cells [26], we asked whether this interaction occurs in our system, including breast cancer and mammalian epithelial cells. Co-immunoprecipitation experiment demonstrated an association between myc-EZH2 and HA-GSK3β in HEK 293T cells (Supplementary Figure S1). Flag-EZH2 and endogenous GSK3β interaction in MDA-MB-231 (Figure 2A) and HA-GSK3β and endogenous EZH2 interaction in HeLa cells (Figure 2B) were observed. In MCF12A cells, endogenous binding of GSK3β to EZH2 interaction was also shown by reciprocal immunoprecipitation (Figure 2C), which is consistent with previous report [26]. EZH2 is predominantly present in the nucleus, whereas GSK3β is found mostly in the cytosol (Figure 2D, right panel). Thus, we further examined the subcellular location of their interaction. Surprisingly, we detected the GSK3β-EZH2 interaction in the cytosol (Figure 2D, left panel). EZH2, indeed, interacted with SUZ12 and EED in the nucleus (Supplementary Figure S2). These results suggested that GSK3β physically interacts with EZH2 in the cytosol.

### GSK3β phosphorylates EZH2

**Figure 3:**
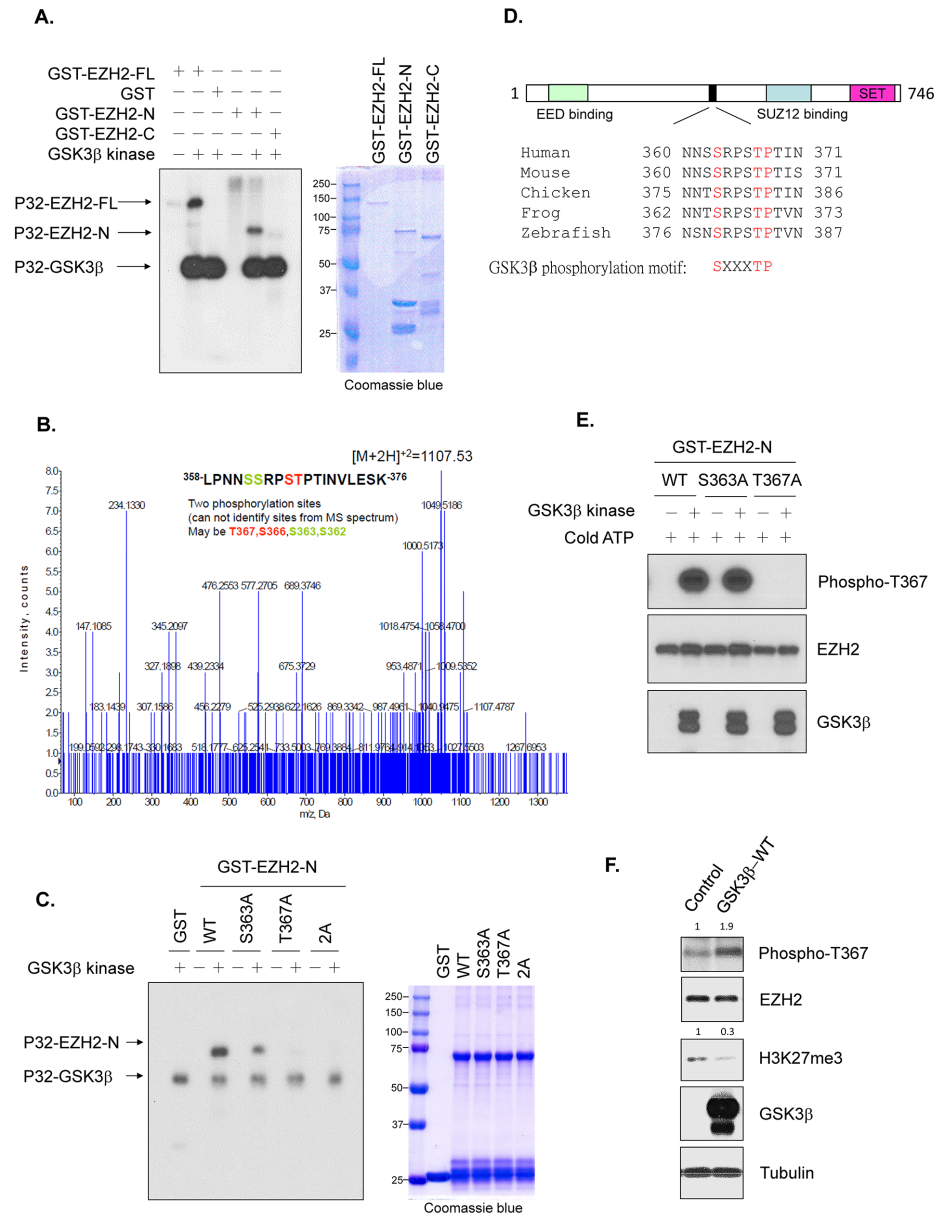
GSK3β phosphorylates EZH2 **A.** *In vitro* kinase assay with recombinant, active GSK3β kinase and full-length GST-EZH2 (FL), GST-EZH2 N-terminal (a.a. 1-385; GST-EZH2-N), or C-terminal (a.a. 386-746; GST-EZH2-C) fragment. Phosphorylation was detected by autoradiography. Loading amount of different EZH2 proteins was accessed by coomassie blue staining. **B.** Mass spectrometry analysis of samples from an *in vitro* kinase assay with GSK3β kinase and GST-EZH2 N-terminal fragment. The spectrum shows that two phosphorylation sites were identified; one was T367 or S366 (marked in red), the other S363 or S362 (marked in green). **C.** *In vitro* kinase assay with active GSK3β kinase and wild-type GST-EZH2 N-terminal fragment (WT), or mutant EZH2 as indicated. Phosphorylation was examined by autoradiography. Loading of EZH2 proteins was assessed by Coomassie blue staining. 2A represents S363A and T367A mutant. **D.** Comparison of GSK3β phosphorylation sites of EZH2 among various species. **E.** Testing of Thr367 phosphorylation antibody using *in vitro* kinase assay with active GSK3β kinase and purified wild-type GST-EZH2-N (WT), GST-EZH2^S363A^-N or GST-EZH2^T367A^-N in the presence of cold ATP at 30°C for 30 min. Reaction mixtures were analyzed by Western blot with mouse serum against Thr367 phosphorylation of EZH2 or antibodies as indicated. **F.** Lysates from cells transfected with control or wild-type GSK3β were immunoblotted with the indicated antibodies. Relative intensities of Thr367 phosphorylation and H3K27me3 bands are shown, normalized to those from cells transfected with control plasmid.

Next, we examined whether GSK3β can phosphorylate EZH2. An *in vitro* kinase assay revealed that GSK3β catalyzed the phosphorylation of EZH2 but not glutathione-S-transferase (GST) (Figure 3A, lanes 1-3). This phosphorylation was catalyzed on EZH2′s N-terminal fragment, rather than its C-terminal fragment (Figure 3A, lanes 4-6). Mass spectrometry analysis using the N-terminal fragment of EZH2 identified 4 possible phosphorylation sites: Ser362, Ser363, Ser366, and Thr367 (Figure 3B). We replaced these 4 residues with alanine individually. An *in vitro* kinase assay demonstrated that the phosphorylation catalyzed by GSK3β was reduced at the Ser363A mutant and was nearly undetectable at the Thr367A mutant (Supplementary Figure S3), which suggests that these two residues are GSK3β phosphorylation sites on EZH2. We further generated a double A (Ser363A & Thr367A) mutant. Consistently, the phosphorylation catalyzed by GSK3β was virtually undetectable (Figure 3C). A search of National Center for Biotechnology Information (NCBI) database using Basic Local Alignment Search Tool (BLAST) revealed that Ser363 and Thr367 of EZH2 are highly conserved among zebrafish to human (Figure 3D).

To confirm the phosphorylation of EZH2 by GSK3β *in vivo*, we generated two specific mouse antisera against the phosphorylated EZH2 at these two residues separately, in which only one antibody could specifically recognize the Thr367 phosphorylation. We validated the specificity of the antibody by an *in vitro* kinase assay using cold ATP. This antibody recognized the GSK3β-catalyzed phosphorylation on wild-type GST-EZH2 or GST-EZH2^S363A^ mutant, but not the non-phosphorylatable GST-EZH2^T367A^ mutant (Figure 3E), suggesting that it can specifically identify Thr367 phosphorylation of EZH2. Using this antibody, we found that ectopic expression of GSK3β enhanced the endogenous level of the phosphorylated EZH2 at Thr367 (Figure 3F). Taken together, these data indicated that GSK3β phosphorylates EZH2 at Ser363 and Thr367.

### GSK3β-mediated phosphorylation of EZH2 downregulates H3K27 trimethylation and EZH2′s oncogenic functions

**Figure 4:**
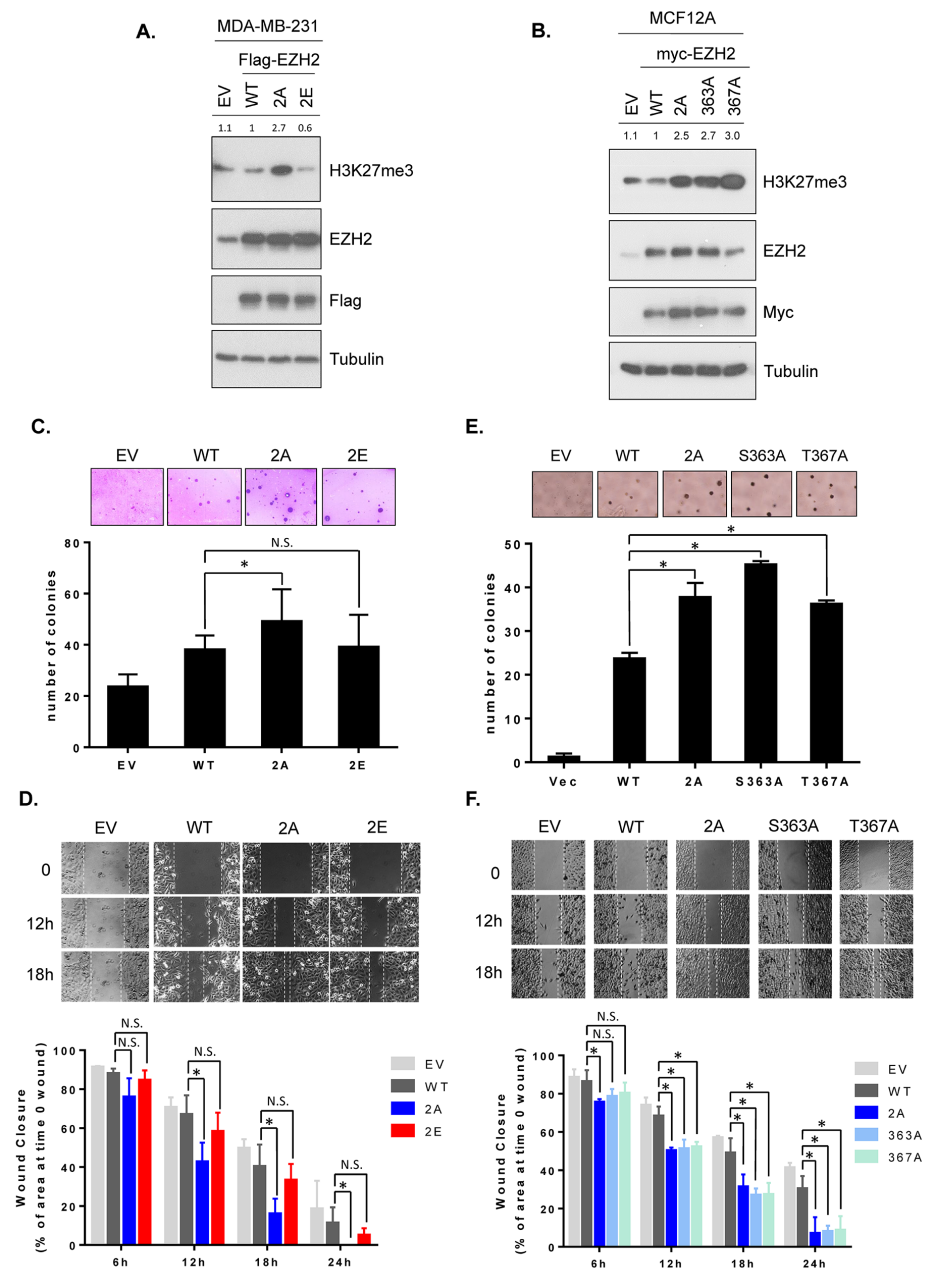
GSK3β-mediated EZH2 phosphorylation decreases H3K27 trimethylation and non-phosphorylatable mutants enhances anchorage-independent growth and cell migration **A.** MDA-MB-231 cells were stably transfected with plasmids encoding wild-type EZH2 (WT), EZH2^2A^ (2A), EZH2 ^2E^ (2E) or empty vector control. Cell lysates were subjected to Western blot analysis with the indicated antibodies. Relative intensities of H3K27me3 bands are shown, compared to those from cells expressing wild-type EZH2. 2E represents Ser363E and Thr367E. **B.** MCF12A cells were stably transfected with plasmids encoding wild-type EZH2 (WT), EZH2^2A^ (2A), EZH2^S363A^, EZH2^S367A^, or control. Cell lysates were immunoblotted with specific antibodies. Relative intensities of H3K27me3 bands are presented. **C.** Colony formation abilities of MDA-MB-231 stable cell lines were determined using soft agar assay. Cells were seeded in 6-well plates as described. The number of colonies counted in one well of 6-well plate is shown as bar graphs. Data are expressed as mean ± s.d. from three independent experiments. Representative images are shown at the top of each bar graph. **D.** Migration potential of MDA-MB-231 stable cell lines were measured by wound healing assay. Cells were seeded in culture inserts and migration was observed by time-lapse microscope as described. Representative images of each line are shown immediately (time 0), 12 h, and 18 h after removal of culture inserts. The areas of wound gap at the indicated time points were determined using the ImageJ software program and normalized to the area of wound gap at time 0. Wound closures were calculated and are plotted as bar graphs. Data are mean ± s.d. from three independent experiments. **E** and **F.** The same experiments as described in C and D were performed in MCF12A stable cell lines. An asterisk (*) indicates a statistically significant difference in the measurements between mutant and wild-type (P < 0.05, Student's *t*-test). N.S., not significant.

To further investigate whether GSK3β phosphorylation sites on EZH2 affects the H3K27 trimethylation and EZH2′s biological functions, we generated stable cell lines expressing the wild-type EZH2, non-phosphorylatable, or phospho-mimic mutants in MDA-MB-231 and MCF12A cells. In MDA-MB-231 cells, we stably transfected with wild-type EZH2, EZH2^2A^ or EZH2^2E^. To examine the effects of individual phosphorylation sites on H3K27 trimethylation levels and EZH2′s functions, MCF12A cells were expressed with EZH2^S363A^, EZH2^T367A^, EZH2^2A^ or wild-type EZH2. We found that EZH2^2A^ increased and EZH2^2E^ decreased H3K27 trimethylation levels (Figure 4A). In MCF12A stable cells, trimethylation of H3K27 was upregulated in all non-phosphorylatable mutants (Figure 4B). Consistently, we observed the similar effects of non-phosphorylatable 2A mutant and phospho-mimic mutants on H3K27 trimethylation levels in MCF7 cells expressing EZH2^2A^, EZH2^S363E^, EZH2^T367E^, or EZH2^2E^ (Supplementary Figure S4). As aforementioned, the association of EZH2 with SUZ12 and EED affects EZH2′s histone methyltransferase activity. However, we did not observe an increased binding of EZH2 to SUZ12 or EED in MCF12A cells expressing non-phosphorylatable EZH2 (Supplementary Figure S5). Collectively, these data suggested that GSKβ-mediated phosphorylation of EZH2 inversely regulates H3K27 trimethylation.

Because EZH2 is known to promote cell transformation [8] and migration [17, 36], and GSK3β-mediated phosphorylation of EZH2 downregulates H3K27 trimethylation, we next studied the effect of non-phosphorylatable and phospho-mimic mutant EZH2 on EZH2-regulated biological functions. Colony formation abilities and cell migration potentials were determined in MDA-MB-231 stably transfected with wild-type EZH2, non-phosphorylatable 2A or phospho-mimic 2E mutant. There were no significant differences in cell proliferation among all transfectants (Supplementary Figure S6). An anchorage-independent soft agar assay revealed that non-phosphorylatable EZH2 (EZH2^2A^) increased anchorage-independent growth more effectively than those of WT EZH2 and phospho-mimic mutant (EZH2^2E^) (Figure 4C). Consistently, a wound healing assay monitored by time-lapse microscopy demonstrated that non-phosphorylatable mutant (EZH2^2A^) enhanced cell migration (faster rate of wound closure) while EZH2^2E^ had similar rate of wound closure, compared to wild-type EZH2 (Figure 4D). To further determine the effect of individual single non-phosphorylatable mutants on the EZH2′s biological functions, we performed a soft agar and a wound healing experiments in noncancerous MCF12A mammary epithelial cells stably expressing EZH2^S363A^, EZH2^T367A^, EZH2^2A^ mutants. The results revealed that all non-phosphorylatable EZH2 promoted anchorage-independent growth as well as cell migration (Figure 4E and 4F). A Boyden chamber migration assay also supported these observations (Supplemental Figure S7). These results suggested that phosphorylation of EZH2 by GSK3β attenuates EZH2′s biological acivities, including anchorage-independent soft agar growth and migration potential.

### H3K27 trimethylation is inversely correlated with GSK3β activity in breast cancer patients

**Figure 5:**
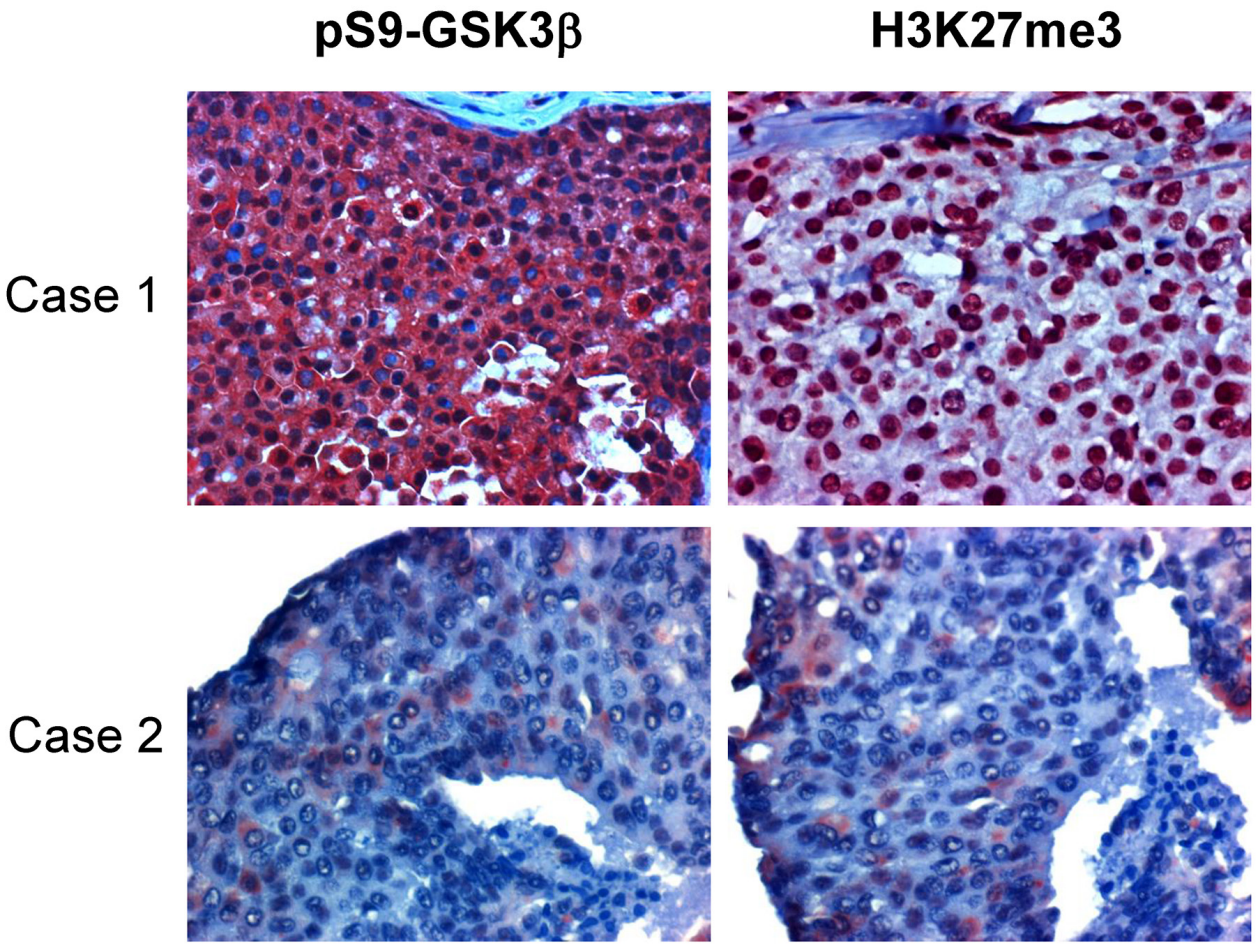
H3K27me3 is correlated with pSer9-GSK3β and low expression of both in tissues associates with better survival in human breast cancers One hundred ten breast tumor tissue samples were subjected to immunohistochemical staining with antibodies specific to phosphorylated GSK3β at Ser9 and H3K27 trimethylation. Representative images shown. Case 1 shows a representative specimen with high expression of Ser9 phosphorylation of GSK3β and H3K27 trimethylation. Case 2 is a sample with low expression of pSer9-GSK3β and H3K27me3.

To examine the pathological relevance of EZH2 regulation by GSK3β, we analyzed correlation between the activity of GSK3β and the enzymatic activity of EZH2 in human breast tumor specimens. Since GSK3β-mediated phosphorylation of EZH2 did not affect EZH2 expression level but reduce H3K27 trimethylation, and it is known that Ser9 phosphorylation can inactivate GSK3β activity and the measurement of Ser9 phosphorylation can be used to determine GSK3β inactivation [25], we compared expression of H3K27 trimethylation with level of GSK3β phosphorylation at Ser9 in tumor tissue samples from 110 breast cancer patients. Consistently, immunohistochemical staining revealed that the level of GSK3β phosphorylation at Ser9 (representing inactivation of GSK3β) was positively correlated with the expression of H3K27 trimethylation (*P* = 0.006; Figure 5 and Table 1). This result suggested that GSK3β activity is inversely related to EZH2 activity in breast cancer tissues.

**Table 1:** Relationship between H3K27 trimethylation (H3K27me3) and pS9-GSK3β expression in human surgical specimens of breast cancer

	H3K27me3			*P* value
	Low	High	Total	
pS9-GSK3β				
Low	13	10	23	
High	23	64	87	
Total	36	74	110	
				0.006

Low: negative (–) and score 1 (+)

High: score 2 (++) and score 3 (+++)

* Correlation between H3K27me3 and pS9-GSK3β was analyzed by the Pearson Chi-Square test. A *P* value < 0. 05 was set as the criterion for statistical significance.

## DISCUSSION

**Figure 6:**
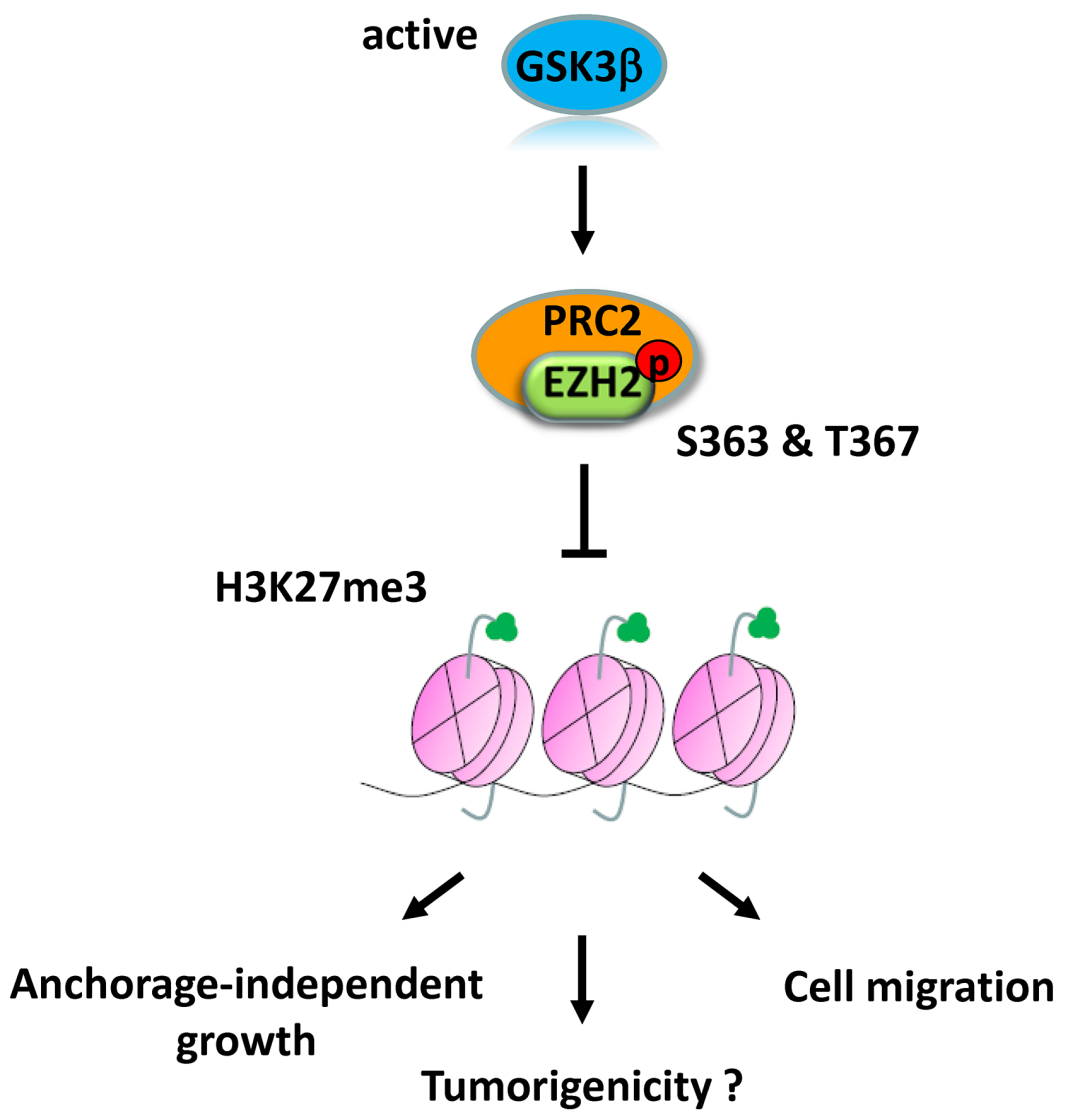
Proposed model of GSK3β-mediated regulation of EZH2 GSK3β phosphorylates EZH2 at Ser363 and Thr367, which suppresses H3K27 trimethylation and EZH2 oncogenic functions.

In the current study, we presented an interesting regulatory mechanism by which GSK3β regulates EZH2 activity via direct phosphorylation (Figure 6). Our study demonstrated that GSK3β physically interacts with and phosphorylates EZH2 at Ser363 and Thr367, without altering EZH2 protein expression, and suppresses H3K27 trimethylation. We found that non-phosphorylatable mutant EZH2 enhanced cell migration and cell growth in an anchorage-independent manner, indicating that GSK3β's regulation is important for the oncogenic functions of EZH2. Consistently, the immunohistochemical staining results revealed that inactivation of GSK3β is significantly correlated with higher level of H3K27 trimethylation in breast cancer patients.

EZH2 has been shown to promotes tumorigenesis in many types of cancers [13, 22]. To prevent tumorigenesis, EZH2 expression and activity need to be critically checked. A recent study reported that GSK3β negatively regulates EZH2 expression in nasopharyngeal cancer cells, and the level of GSK3β phosphorylation at Ser9 is associated with higher EZH2 protein expression in patients with nasopharyngeal carcinoma [26]. In our study, we found that GSK3β negatively regulates EZH2′s enzymatic activity, which is reflected by the expression of H3K27 trimethylation, without altering EZH2 protein level, and this observation was further supported by our immunohistochemical staining analysis in human breast cancer tissue samples. Although the previous study and our findings suggest that EZH2 is subjected to GSK3β control, the underlying mechanism of this regulation may differ in different cancer types.

GSK3β is known to be involved in tumor development, but its role in tumorigenesis remains controversial. In some types of cancer like pancreatic and ovarian cancers, it contributes to tumorigenesis; in others such as breast cancer and nasopharyngeal carcinoma, it functions as a tumor suppressor [30, 37]. In our study, we demonstrated that GSK3β non-phosphorylatable mutant EZH2 (EZH2^2A^, EZH2^S363A^, EZH2^T367A^) enhances cell migration and anchorage-independent growth in breast cancer and mammary epithelial cells. Together with previously published reports, our results further strengthened the role of GSK3β as a tumor suppressor in breast cancer and implied that inactivation of GSK3β is one of the mechanisms enhancing EZH2 activity in cancer.

Studies have shown that EZH2 can be translationally modified by phosphorylation [14–22]. Previous phosphoproteomic analyses of EZH2 have identified many phosphorylation residues in mouse tissues and human cell lines, including Ser362, Ser363, Ser366 and Thr367 [38, 39]. Our work confirmed that GSK3β phosphorylates EZH2 at Ser363 and Thr367. As mentioned earlier, GSK3β has a preference for its substrates by recognizing a phosphorylation motif. Interestingly, the identified phosphorylation sites on EZH2 are compatible with this consensus motif. Furthermore, these two phosphorylation sites are highly conserved residues, implying that this regulation could be functionally important in other organisms. We attempted to determine the *in vivo* relationship between GSK3β and phosphorylated EZH2 in human tumor samples by using antibodies against these phosphorylation sites, but the antibodies we generated did not work for immunohistochemical staining. Previously, a study revealed that EZH2 can be phosphorylated at Thr367 by p38α in muscle stem cells [20]. p38α-mediated EZH2 phosphorylation leads to *Pax7* repression through trimethylation of H3K27 in its promoter while inhibition of p38α-EZH2 pathway promotes muscle stem cell proliferation. In our study, we observed that non-phosphorylatable mutant, EZH2^T367A^, increased global H3K27 trimethylation in mammary epithelial cells and enhanced cell growth and migration. Activation of GSK3β upregulated Thr367 phosphorylation. Our findings and previous report suggested that the biological significance of EZH2 phosphorylation at Thr367 is context-dependent.

The histone methyltransferase activity of EZH2 can be regulated by site-specific phosphorylations in different mechanisms. For example, JAK2 phosphorylates EZH2 at Tyr641, which is located on the catalytic SET domain, promotes EZH2′s interaction with β-TrCP and leads to its degradation [21]. Thr487 phosphorylation by CDK1 disrupts PRC2 assembly and reduces H3K27 trimethylation [17]. In the present study, these two phosphorylation sites we identified are neither located on the SET domain nor in the regions of SUZ12 or EED binding. Compatible with the observation, we (Supplemental Figure S4) and others [20] did not find that mutations at these sites (EZH2^2A^, EZH2^S363A^, EZH2^T367A^) change the association of EZH2 with EED or SUZ12. Furthermore, we did not detect alteration in EZH2 protein expression upon this regulation. However, our work revealed that GSK3β interacts with EZH2 and, unexpectedly, their interaction is in the cytosol. Previous report also showed that EZH2 has a cytosolic role [40]. Thus, we hypothesized that GSK3β regulates H3K27 trimethylation by mediating EZH2′s localization. Further investigation is needed to verify this hypothesis. Yet, other possibility can not be excluded.

EZH2 has been shown to enhance WNT/β-catenin pathway by downregulating CXC finger protein 4 [37], and our previous work also indicated that EZH2 represses *RAD51* expression which in turn activates β-catenin signaling [9]. β-catenin is a critical substrate of GSK3β, and aberrant GSK3β/β-catenin activity has been demonstrated to play a key role in tumorigenesis [31]. Inhibitors targeting EZH2 have been developed and tested in clinical trials [41]. Notably, the use of EZH2 inhibitor in colon cancer cells was reported to restore WNT/β-catenin activity [13]. This regulation of EZH2 by GSK3β directly links epigenetic modulation to the WNT/β-catenin pathway. Moreover, an interesting study reported that specific KRAS mutation regulates EZH2 protein expression through the PI3K/AKT and/or MEK/ERK signaling pathways in lung cancer [42]. EZH2 inhibition enhances the sensitivity to MEK-ERK or PI3K/AKT targeted therapies in specific KRAS-mutant lung cancer cells and tumors. Since GSK3β is known to be inactivated by AKT or ERK [25, 43], our work suggests a direct role of the GSK3β-EZH2 pathway in this scenario and offers a rationale for enhancing GSK3β activity and/or targeting EZH2 in anti-cancer therapy.

## MATERIALS AND METHODS

### Cell culture

All cell lines were obtained from ATCC (Manassas, VA) and their validation was performed in Characterized Cell Line Core Facility, MD Anderson Cancer Center. The cell lines used in this study include MDA-MB-231, BT549, MDA-MB-435S, MDA-MB-468, MCF7, MCF12A, HeLa and 293T cells. All cells except MCF12A were grown in Dulbecco's Modified Eagle's Medium/F12 supplement (DMEM/F12) supplemented with 10% heat inactivated fetal bovine serum (FBS), Penicillin/Streptomycin (100 U, 100 μg/ml) at 37°C in a humidified atmosphere with 5% CO2. MCF12A cells were cultured in DMEM/F12 media supplemented with 5% horse serum. Transfection of cells with DNA was performed with liposome. EZH2 stable transfectants and GSK3β stable knockdown by shRNA were selected by either G418 (Fisher) or puromycin (Invivogen). For inhibiting or activating GSK3β activity, cells were treated with 40 mM lithium chloride (Sigma) for 16 h or 0.1 uM staurosporine (Sigma) for 2 h.

### Western blot, immunoprecipitation and qRT-PCR

Whole cell lysis, subcellular fractionation, western blot and immunoprecipitation were performed as described [17, 44]. Quantification of the band density of target proteins in western blot experiments was analyzed by free software ImageJ. The following antibodies were used in western blotting and immunoprecipitation: EZH2, phospho-GSK3β Ser9, trimethyl-H3K27, and histone H3 (Cell Signaling Technology); GSK3β (BD Biosciences); dephospho-β-catenin (Ezno Life Sciences); Tubulin and Actin (Sigma); Lamin B1 (Abcam); Myc and HA (Roche). The mouse phospho-EZH2 Thr367 antibody was produced against the synthetic peptides SRPS(pT)PTINVLESKD at China Medical University Hospital in Taiwan. The synthetic peptides were obtained from LifeTein LLC. Total RNA was extracted from cells using TRIzol. Quantitative real-time PCR (qRT-PCR) was performed using SYBR Green dye on a Bio-Rad PCR machine. The primer sequences used for analysis of HOXA gene expression was listed in Supplementary Information Table S1.

### Plasmids

Plasmids of HA-GSK3β-WT (wild-type), HA-GSK3β-CA (constitutively active, S9A GSK3β), and HA-GSK3β-KD (kinase dead, K85R GSK3β) were described previously [29]. pCDNA3-Myc-EZH2 was a gift from A. Chinnaiyan. For EZH2 stable transfection, two EZH2 constructs were generated. Myc-EZH2 was subcloned into the vector of pCDH-CMV-MCS-EF1-Puro (System Biosciences), and Flag-EZH2 was subcloned into the vector of pCDH-CMV-MCS-EF1-Neo (System Biosciences). To generate constructs for bacterial expression of GST-tagged EZH2, two truncations were made in GST-N terminal fusion protein format in pGEX-6P1 vector (Amersham Biosciences or GE Healthcare). One EZH2 truncation, N-terminal fragment, was from amino acid residues 1-385, and another, C-terminal fragment, was from 386-746. Site-directed mutagenesis was performed to generate mutant EZH2 according to a protocol [45]. Primers used for mutagenesis were provided in Supplemental Information Table S2. GSK3β knockdown was carried out by pGIPZ-shRNA with the target sequence of 5′-TACTTGACAGTTCTTGAGT-3′ (CDS, Clone ID V3LHS_309039, shRNA core facility, MD Anderson Cancer Center).

### *In vitro* kinase assay

Recombinant, active GSK3β kinase was obtained from Life Technologies and glutathione-S-transferase (GST)-fused full length EZH2 was purchased from BPS Bioscience. GST-EZH2 C-terminal, wild-type and mutant GST-EZH2 N-terminal fragments were purified from bacteria. For the GSK3β *in vitro* kinase assay, active GSK3β kinase was incubated with wild-type or mutant GST-EZH2 purified proteins in kinase buffer (50 mM Tris-HCl at pH 7.6, 10 mM MgCl_2_, 2 mM DTT and 0.1 mM EDTA) in the presence of 5 μCi of [γ-^32^P]ATP and 50 μM cold ATP with substrates at 30 °C for 30 min [46]. Reaction mixtures were then subjected to SDS-PAGE, and ^32^P-labelled proteins were detected by autoradiography.

### Mass spectrometry

To identify phosphorylation sites of EZH2, mass spectrometry analysis was performed as previously described [17]. Briefly, GST-fused EZH2 purified protein was incubated with GSK3β kinase in a kinase reaction mixture at 30 °C for 30 min. After being resolved by SDS-PAGE, the protein band corresponding to EZH2 was excised and subjected to digestion with trypsin. The phosphopeptides were then isolated by immobilized metal affinity chromatography. The micro-liquid chromatography/tandem mass spectrometry (LC–MS/MS) was performed to analyze the phosphopeptides and to identify phosphorylation sites of EZH2.

### Soft agar and wound healing assays

For the soft agar transformation assay, 2.5 × 10^4^ cells were seeded in 1 ml of regular medium with 0.5% low melting point agarose and overlaid on 1 ml of medium with 1% agarose in each well of a six-well plate. After 3 weeks, colonies larger than 100 μm in diameter were counted. The wound healing assay was performed using a culture-insert (ibidi GmbH, Germany) according to manufacturer's instruction. The culture-insert had two cell culture reservoirs, which were separated by a 500 μm-thick wall. Same numbers of cells were seeded in the culture-insert. After 24 h, the culture-insert was removed, which left a cell-free “wound” of around 500 μm in width. The wound closure was observed by a time lapse microscopy (Zeiss, Germany) and images were obtained at 1 h interval for 24 h. The area of wound was analyzed using the ImageJ software program.

### Immunohistochemical staining

Human breast tumor tissue samples were obtained following the guidelines approved by the Institutional Review Board of MD Anderson Cancer and written informed consents were obtained from all patients at the time of enrollment. Immunohistochemical staining was performed in 110 human breast cancer tissue specimens obtained from the Department of Pathology, Shanghai East Breast Disease Hospital, Shanghai, P.R. China. Each specimen was stained with specific antibodies against pS9-GSK3β (Cell Signaling Technology) and H3K27 trimethylation (EMD Millipore) and scored by an H-score method as previously described [47]. The intensity of staining was ranked into four groups: high (score 3), medium (score 2), low (score 1), and negative (score 0). High and medium intensity of staining were grouped into “high expression”. Low and negative were “low expression”.

### Statistical analysis

All data were representative of at least three independent experiments. All P values were analyzed by student's *t*-test except for calculation of correlation analysis. For correlation analysis, a Pearson chi square test (SPSS software) was used to examine the relation between pS9-GSK3β expression and H3K27 trimethylation levels. A P value < 0.05 was considered statistically significant.

## SUPPLEMENTARY MATERIALS FIGURES AND TABLES


