# Supplementary material for: GSK3β inactivation promotes the oncogenic functions of EZH2 and enhances methylation of H3K27 in human breast cancers
Source: Oncotarget. 2016 Aug 2;7(35):57131–44. doi: 10.18632/oncotarget.11008 (PMC5302978; doi:10.18632/oncotarget.11008)
Supplement: Supplementary file 1 [file oncotarget-07-57131-s001.pdf]

GSK3β inactivation promotes the oncogenic functions of EZH2 and enhances methylation of H3K27 in human breast cancers

SUPPLEMENTARY FIGURES AND TABLES

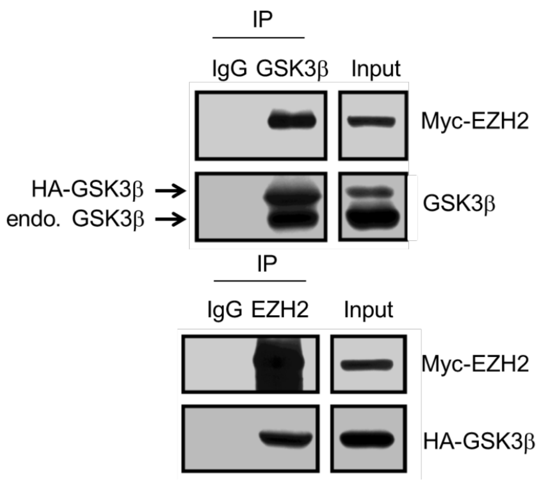

Supplementary Figure S1: GSK3β interacts with EZH2. 293T cells were transfected with plasmids encoding Myc-EZH2 and HA-GSK3β. Cell lysates were immunoprecipitated with HA (upper panel) or Myc (lower panel) antibodies, followed by Western blot analysis to detect Myc-EZH2, HA-tag and endogenous GSK3β as indicated. Immunocipitation with immunoglobulin (IgG) served as control.

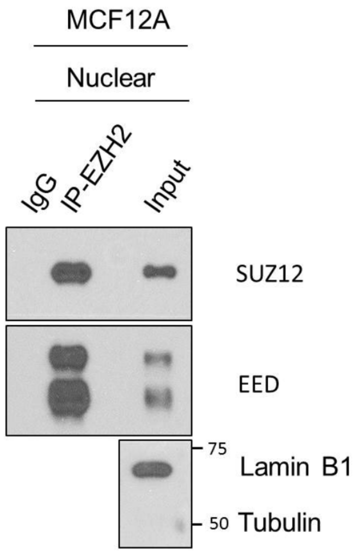

Supplementary Figure S2: EZH2 associates with SUZ12 and EED in the nucleus. MCF12A cells were lysed and followed by cellular fractionation. Nuclear fraction was immunoprecipitated with EZH2 antibody and immunoblotted by antibodies against SUZ12 and EED. Lamin B1 and tubulin were used as markers for nuclear and cytosolic fractions, respectively.

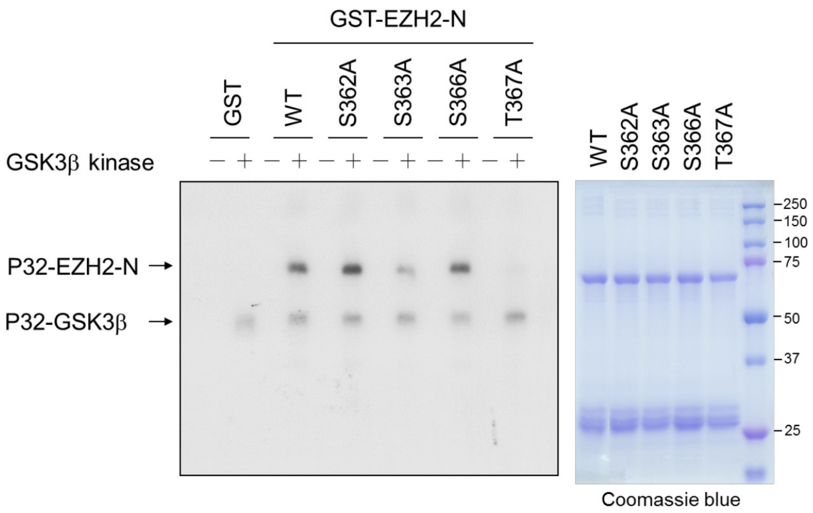

Supplementary Figure S3: GSK3β phosphorylates EZH2 at Ser363 and Thr367. *In vitro* kinase assay with active GSK3β kinase and wild-type GST-EZH2 N-terminal fragment (WT), $EZH2^{S362A}$-N $EZH2^{S363A}$-N, $EZH2^{S366A}$-N, or $EZH2^{T367A}$-N. Phosphorylation was examined by autoradiography. Loading of GST-EZH2 N-fragment was assessed by Coomassie blue staining.

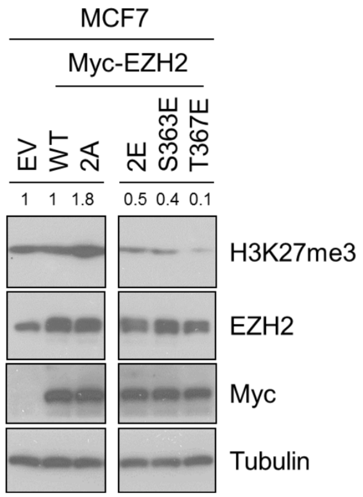

Supplementary Figure S4: GSK3β-mediated EZH2 phosphorylation negatively regulates H3K27 trimethylation. MCF7 cells were stably transfected with plasmids encoding wild-type EZH2 (WT), $EZH2^{2A}$ (2A), $EZH2^{2E}$ (2E), $EZH2^{S363E}$, $EZH2^{T367E}$, or vector control. Cell lysates were subjected to Western blot analysis using the indicated antibodies. Relative intensities of H3K27me3 bands are shown, compared to those from cells expressing wild-type EZH2. 2E represents Ser363E and Thr367E.

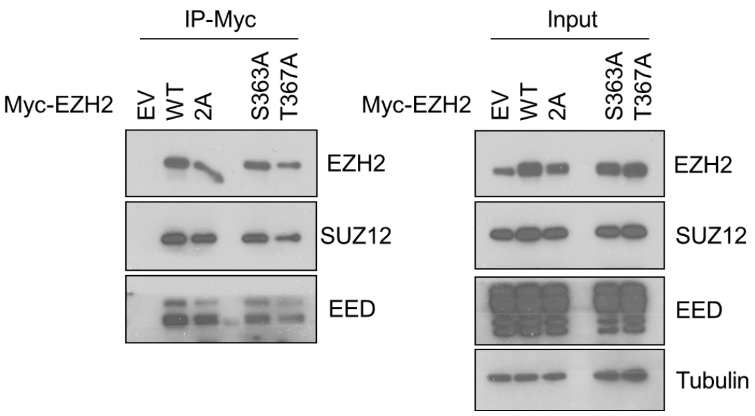

Supplementary Figure S5: GSK3β-mediated EZH2 phosphorylation does not affect the association of EZH2 with SUZ12 and EED. Cell lysates from MCF12A stable cells expressing wild-type EZH2 (WT), $EZH2^{2A}$ (2A), $EZH2^{S363A}$, $EZH2^{T367A}$, or vector control were immunoprecipitated with Myc antibody, followed by immunoblotting with EZH2, SUZ12 and EED antibodies (left panel). Input lysate was analyzed to detect the expression levels of the indicated proteins (right panel).

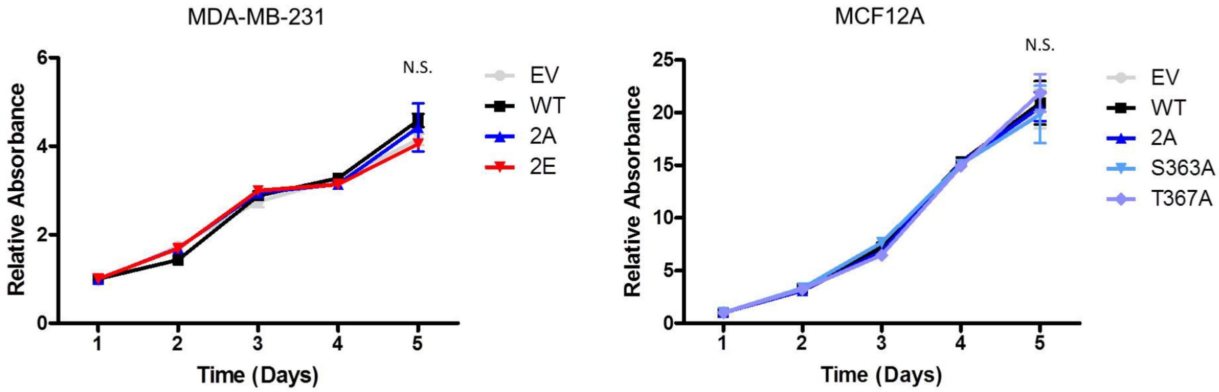

Supplementary Figure S6: GSK3β-mediated EZH2 phosphorylation does not affect cell proliferation. Cell proliferation of MDA-MB-231 and MCF12A stable cell were measured by MTT assay. Cells were seeded in 96-well plates at a density of 2×10^3 cells per well. At each time point, cells were stained with 100 µL of sterile MTT (1 mg/mL; Sigma) for 4 hours at 37 °C, followed by removal of the culture medium and addition of 100 µL of dimethyl sulfoxide. Absorbance was measured at 570 nm, using 655 nm as the reference wavelength. All experiments were carried out in triplicate. N.S., no statistical difference in the measurements between each mutant and wild-type at the last time point.

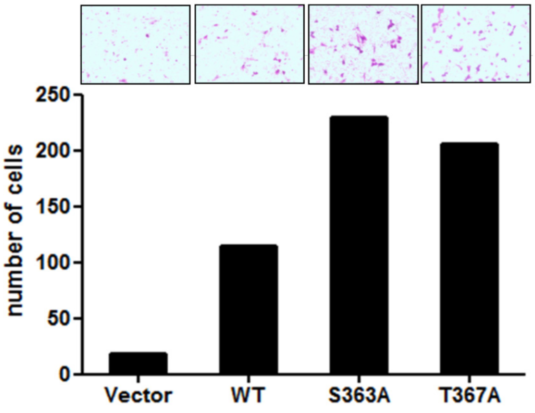

Supplementary Figure S7: GSK3β-non-phosphorylatable mutant EZH2 enhances cell migration. Migration abilities of MCF12A cells expressing wild-type EZH2 (WT), $EZH2^{S363A}$, $EZH2^{T367A}$, or vector control were determined using 24-well Boyden chamber plates with an 8-µm pore size polycarbonate filter (BD Biosciences). 1×10^5 cells were seeded into upper chambers with serum-free medium. Lower chambers contained 10% FBS medium. After incubation at 37 °C for 24 hours, chamber filters were fixed and stained. Cells on the top of filters were removed. The number of migrated cells was counted under a light microscopy and shown as bar graph. Representative images are shown at the top.

Supplementary Table S1: Primers for RT-PCR

Gene name	Forward primer	Reverse primer
HOXA2	ACAGCGAAGGGAAATGTAAAAGC	GGGCCCCAGAGACGCTAA
HOXA3	TGCAAAAAGCGACCTACTACGA	CGTCGGCGCCCAAAG
HOXA7	CAAAATGCCGAGCCGACTT	TAGCCGGACGCAAAGGG
HOXA9	CCGAGAGGCAGGTCAAGATC	AAATAAGCCCAAATGGCATCA
HOXA13	AAATGTACTGCCCCAAAGAGCA	ATCCGAGGGATGGGAGACC
GAPDH	TCCACTGGCGTCTTCACC	GGCAGAGATGATGACCCTTTT

Supplementary Table S2: Primers for mutagenesis

Target	Forward primer	Reverse primer
S362A	CAATAACGCTAGCAGGCCCAGCACCCCCAC	CCTGCTAGCGTTATTG<br>GGAAGCCGTCCTCTTC
S363A	CAATAACAGTGCCAGGCCCAGCACCCCCAC	CCTGGCACTGTTATTG<br>GGAAGCCGTCCTCTTC
S366A	CCCGCCACCCCCACCATTAATGTGCTGGAATC	GGGGGTGGCGGGCC<br>TGCTACTGTTATTGGG
T367A	CCCAGCGCCCCCACCATTAATGTGCTGGAATC	GGGGGCGCTGGGCCT<br>GCTACTGTTATTGGG
2A	GCCAGGCCCAGCGCCCCCACCATTAATGTGCTG	GGCGCTGGGCCTGGCACT<br>GTTATTGGGAAGCCGTC
S363E	CAATAACAGTGAAAGGCCCAGCACCCC	CCTTTCACTGTTA<br>TTGGGAAGCCGT
T367E	CCCAGCGAACCCACCATTAATGTGCTG	GGGTTCGCTGGGCCTGCTACTG
2E	GAAAGGCCCAGCGAACCCACCATTAATGTGCTGG	TTCGCTGGGCCTTTCAC<br>TGTTATTGGGAAGCCGTC
